# Navigating tensions between scientific advancement and ethical practice in biobanking: a qualitative study in Japan

**DOI:** 10.3389/fgene.2026.1778587

**Published:** 2026-03-10

**Authors:** Masanori Oikawa, Yoshiyuki Takimoto

**Affiliations:** 1 Department of Medical Ethics, Tohoku University Graduate School of Medicine, Sendai, Japan; 2 Department of Medical Ethics, Kobe University Graduate School of Medicine, Kobe, Japan

**Keywords:** biobank, ethics, governance, interview, Japan, qualitative study

## Abstract

The long-term sustainability of biobanking depends on governance systems that are both ethically robust and operationally reliable. Despite this importance, only a limited number of empirical studies have examined which governance policies are actually implemented in biobank operations and what challenges arise in practice. This lack of systematic research is particularly evident in Japan. To address this gap, we conducted semi-structured interviews with professionals involved in the operation and management of four Japanese biobanks. The study aimed to elucidate the underlying concepts and policies of organizational governance, as well as the challenges encountered in their implementation. The analysis generated three main themes: (a) organizational management and governance; (b) expansion of the scope of utilization and improvement of utilization rates and efficiency; and (c) stakeholders and research governance. These themes reflect the tension inherent in biobanks as medical research infrastructure between scientific advancement and ethical practice. It was within this tension that governance was conceived and enacted in everyday practice.

## Introduction

With the advancement of genomic and precision medicine, biobanks have become an essential research infrastructure for the long-term storage and use of biological samples and associated data (Coppola et al., 2019). Because biobanks are designed to operate over extended periods of time, their sustainability depends not only on scientific and technical capacity but also on governance systems that are both ethically robust and operationally reliable (Chalmers et al., 2016; Samuel et al., 2022b; Simeon-Dubach and Watson, 2014). While early bioethical debates on biobanking primarily addressed normative issues such as consent models, privacy, and ownership, a growing body of empirical research has shifted attention toward how these norms are interpreted and enacted in the operational settings (Hoeyer, 2008).

In particular, qualitative studies examining the perspectives of various stakeholders—such as researchers, policymakers, members of institutional review boards, donors, patients, and citizens—suggest that biobank governance is shaped not only by normative frameworks but also by a range of practical contexts (Haddow et al., 2007; Heredia et al., 2017; Hoeyer and Tutton, 2005; Lemke et al., 2010; Matandika et al., 2020; Van der Velden et al., 2023; Yip et al., 2019). Operational decision-making in biobanks is often carried out through interpretive and context-dependent processes that take into account research objectives, resource constraints, and regional cultural and historical contexts.

More specifically, qualitative studies indicate that institutional decisions regarding the implementation of broad consent, data sharing, commercialization, and the return of results are influenced by considerations of stakeholder acceptability and trust. Biobank operations often involve pragmatic adjustments to align institutional policies with socially acceptable practices and organizational norms (Hoeyer and Tutton, 2005). Qualitative research has been particularly useful in elucidating how biobank professionals collectively shape the substance of biobank governance.

Despite these insights, empirical qualitative studies that systematically examine the governance from the perspectives of internal actors in Japanese biobanks remain limited. Generating empirically grounded knowledge within the Japanese institutional and cultural context is therefore essential for advancing discussions on the ethical and operational sustainability of biobank governance.

In Japan, biobank research has been promoted as part of national research and development strategies, while its operation and governance are regulated through a combination of laws and ethical guidelines rather than a single comprehensive biobank law. In particular, the *Ethical Guidelines for Medical and Biological Research Involving Human Subjects* provide a general framework for the collection and use of biological samples and data, including requirements related to consent, secondary use, anonymization, and information disclosure. However, the practical interpretation and implementation of these guidelines are largely delegated to individual research institutions and biobanks. Compared with many Western countries, Japan lacks a dedicated legislative framework specifically governing biobanks, resulting in a regulatory environment in which governance practices are shaped through the interpretation of existing guidelines and data protection regulations. While this institutional arrangement allows a certain degree of flexibility in biobank operations, it also creates room for variation across institutions in how the governance policies are understood and enacted in practice. Examining how biobank professionals navigate and operationalize these norms within the Japanese institutional context is therefore of particular relevance to international discussions on biobank governance.

The aim of this study is to explore how biobank governance is interpreted and implemented in practice by biobank managers in Japan through a qualitative interview study. Specifically, this study seeks to examine (1) how institutional policies related to governance are translated into the operational practices, and (2) what kinds of challenges, tensions, and uncertainties arise in the course of this implementation. By providing empirically grounded insights from the Japanese institutional and cultural context, this study aims to contribute to the international literature on biobank governance and to inform future discussions on ethically robust and operationally sustainable governance frameworks.

## Methods

### Sampling procedure

The inclusion criteria for biobanks in this interview study were that they have a minimum level of infrastructure and functionality to collect, store, and distribute biospecimens and associated data for research purposes. To capture qualitative data on diverse aspects of biobanking in Japan, the study sample was designed to include at least one biobank from each of the following categories: a large population-based biobank, a small-to medium-sized biobank, and a disease-specific biobank. In addition, to achieve the objectives of this study, we focused on biobanks that had been established for several years, publicly disclosed information on their websites, and demonstrated an adequate level of governance in their management and operations. However, at the time the study was designed, only a limited number of biobanks met these criteria. Consequently, this exploratory study adopted a purposive sampling approach, and five institutions in Japan were selected for participation.

The inclusion criteria for participants at each institution were individuals who were involved in the operation and governance of the biobank. Potential participants were identified through the authors’ research networks and were invited to participate in the study via e-mail. Four of the five individuals contacted responded to the invitation and agreed to participate in the study (IDs: PA, PB, PC, and PD1). In addition, one participant (PD1) introduced two other eligible members from the same institution (PD2 and PD3). As a result, a group interview was conducted at that institution. Although data saturation was not used as a criterion for determining sample size *a priori*, analyses indicated that no substantively new themes emerged by the fourth interview, suggesting that data saturation was achieved with respect to the study objectives.

### Interviews

One author (MO) conducted semi-structured online interviews between January and February 2021. The interviews were designed to elicit a broad range of perspectives on biobank operations and governance. Participants were invited to speak freely about their institution’s operational policies, the processes through which these policies were developed, related concerns, and future directions. To facilitate open and active discussion, a set of key terms related to ethics and governance was prepared in advance and introduced as open-ended prompts when appropriate (Table 1). These key terms were identified through a literature review on ethical and social issues in biobanking conducted prior to the study. The appropriateness of the interview design was ensured through iterative discussions among the co-author. All interviews were audio-recorded and lasted approximately 90 min. Intelligent verbatim transcripts were prepared for subsequent analysis.

**TABLE 1:** Interview topics and guiding questions.

**Background to current operational policies** **Q**. Could you describe your biobank and its current operations? How were the current operational policies and governance arrangements developed, and through what processes? ***Keywords***: research purpose, types of biospecimens and data collected, periods of storage and use of biospecimens and data, involvement of commercial entities, collaboration with other institutions, return of individual research results **Q**. What considerations informed these decisions? ***Keywords*** *:* donor’ perspectives and feelings, respect for autonomy, sustainability of the biobank, transparency, accountability, alternative consent models
**Concerns encountered in biobank operations** **Q**. What concerns have arisen to date, and how have they been addressed? ***Keywords*** *:* donor recruitment, consent procedures, withdrawal of consent, information disclosure
**Future directions** **Q**. What are your views on the future direction of the biobank’s operational policies?
*In addition, to encourage participants to articulate their views, the following key terms were prepared in advance and used flexibly as open-ended prompts: Right to know or not to know Personal data and privacy, discrimination, stigma, and potential cultural harms Access to and control over biospecimens and data Perceived social value or contribution Financial incentives or other forms of compensation for donors

### Analysis

The transcripts were analyzed using inductive thematic analysis (Boyatzis, 1998; Braun and Clarke, 2006; Nowell et al., 2017; Tsuchiya, 2016). Thematic analysis is a qualitative research method that can be applied across a wide range of research questions and data types to identify, analyze, and report patterns or themes within a dataset. It is particularly useful for exploring participants’ perspectives, examining similarities and differences across accounts, and generating insights that may not have been anticipated at the outset of the study. Owing to its theoretical flexibility, thematic analysis offers a robust and adaptable approach for producing rich, detailed, and nuanced interpretations of qualitative data (Braun and Clarke, 2006; Nowell et al., 2017).

First, the author (MO) coded the transcript of the interview with PA. The transcript of PB was then coded using the existing code set, with new codes added as necessary. This iterative process was subsequently applied to the transcripts of PC and PD (including PD1, PD2, and PD3) in the same manner. Throughout the coding process, codes were refined, merged, or revised as appropriate, and their validity was repeatedly examined across all transcripts.

Second, conceptually related codes were grouped into broader categories. Third, themes were developed and identified to capture the underlying concepts and logic of biobank operations. Finally, the consistency of the analytic process, as well as the identified codes and themes, was reviewed and confirmed by a co-author (YT) with expertise in qualitative methodology. Coding was conducted using NVivo (Release 1.6; QSR International Inc.).

## Results

### Participants’ characteristics

Table 2 shows the characteristics of participants and information about their institutions.

**TABLE 2:** Participants’ characteristics.

Participant ID	Institution/Biobank type	Participants’ role in the institution
PA	Mid-sized	Risk-management
PB	Disease-specific	Administration and planning
PC	Large population	Management
PD	Small-sized	​
PD1	​	Administration of biospecimens
PD2	​	Management
PD3	​	Management for basic research support department

#### Themes

Table 3 shows the codes, categories, and themes that emerged from the data analysis. Three main themes were found: (a) organizational management and governance; (b) expansion of the scope of utilization and improvement of utilization rate and efficiency; and (c) stakeholders and research governance.

- Organizational management and governance

**TABLE 3:** Themes and categories.

Theme
Category
Organizational management and governance
Risk management
Quality management
Finance
Expansion of the scope of utilization and improvement of utilization rate and efficiency
Utilization
Efficiency
Stakeholders and research governance
Public perceptions and relationship-building
Medicine and health of the public, and relationship-building
Relationship-building with local government and medical associations
Relationship-building with patient advocacy groups and family associations
Donors and research governance
Family and research governance

The main categories in this theme included risk and quality management and finance, which, in most cases, were described as direct factors contributing to the improvement of organizational governance in biobanks.

The risks identified included information leakage and the commodification of biospecimens. These risks were described as affecting not only donors but also the organizations themselves, as they may constitute legal breaches and significantly undermine public trust. Because of their potential impact on the organizations, these issues were often discussed in relation to organizational governance. At the same time, information leakage and commodification were also understood as raising ethical concerns related to donor privacy, dignity, and the appropriate use of biospecimens. Consideration of how to address these ethical concerns appeared to inform governance approaches, while the issues themselves were likewise framed as matters of organizational governance.

The information risk is addressed if the specimens are properly anonymized so that when they arrive here and are stored, there is no name on the specimen label and only the specimen number reassigned by us is listed. Even if someone simply stole it, there would be no name on it. Therefore, information risk is handled through proper anonymization (PA).

The on-demand type of distribution is most closely related to laws regulating organ procurement and trafficking. The reason for this is that we receive money for necessary expenses even though it does not represent a profit; thus, taking a human specimen and selling it as it is, and making a profit, is creating a commodity (PD2).

In the latter discourse, on-demand distribution—which relates to theme (b) discussed below—was described as the acquisition and provision of biospecimens and associated data in response to requests from researchers or commercial entities in order to enhance their utility. Within the Japanese context, where the commercial trade of organs and human tissues is legally prohibited, the participant reported internal discussions about whether charging necessary costs when initiating on-demand provision could potentially conflict with existing legal restrictions, and noted that these discussions also addressed how governance policies should be formulated at the institutional level in response to this concern.

The importance of international standardization of procedures in quality management was emphasized. This was understood not only in scientific terms, as ensuring the quality of biospecimens and associated data, but also as contributing to the proper operation of the biobank itself. Such efforts were viewed as supporting robust governance practices, promoting harmonization with international standards and practices, and benefiting a wide range of stakeholders, thereby contributing to the long-term sustainability of the biobank.

In a word, international standardization is the most important aim now. International standardization has scientific merit, but it also is important for the users or stakeholders. […] We are now preparing to properly operate in accordance with the quality management system, where operated individually and the governance has not been sufficient, and to ensure sustainability by doing so (PB).

Some of the participants reported concerns about financial security as a basis of organization. One participant noted that while the government provided funding assistance when the biobank was established, this would not necessarily continue indefinitely. Another participant revealed that organizational decisions are influenced by financial circumstances:

The main objective is to stimulate research within the university, and we provide samples [outside the university] just to run the various costs involved. […] I think the reality is that we have made several [governance] decisions based on the need to reduce running costs (PD2).

This participant was a manager at an institution implementing the on-demand distribution described above. The decision to introduce on-demand provision, alongside external sharing of biospecimens and associated data, was reported to have been influenced in part by financial considerations. This suggests that financial considerations may play a role in shaping operational policies within biobanks. While external sharing and on-demand provision can enhance convenience for researchers and industry partners and thereby support scientific advancement, they may also raise ethical concerns, including whether adequate understanding and acceptance can be obtained from donors and the public, particularly when financial incentives are involved. The fact that such policies may be influenced by financial considerations therefore has significant implications.

b. Expansion of the scope of utilization and improvement of utilization rate and efficiency

This theme captured issues related to use and efficiency as they pertain to the primary purposes of biobank activities. Discussions concerning the utilization of biospecimens and data encompassed a range of approaches implemented by each biobank to contribute to the advancement of medicine. All participating institutions made their collections available to external researchers, including those from commercial entities, and one biobank also provided access to overseas researchers. As described above, another institution introduced on-demand distribution tailored to the needs of commercial partners as a strategy to expand utilization while also addressing financial concerns.

Commercial use, in particular, was regarded as an essential component of biobanking, for expanding the scope of utilization and increasing rates of use:

On the other hand, it is very attractive for companies and research institutions that normally have difficulty accessing specimens linked to clinical information, so that type of utilization contributes to the overall utilization rate (PA).

However, all participants expressed some concern about commercial uses and reported challenges in establishing effective and robust systems to manage them.

When we were banking spinal fluid, as I mentioned earlier, we used a multiple-choice consent form on whether or not to provide it to a company. At that time, the ethics committee was very strict about, for example, providing it to a company (PB).

There are people who do not want the samples that they gave to be used by companies, and I wondered how I could respond to that kind of need, and if it would be possible to create a system (PC).

With respect to these robust systems, one biobank adopted a more restrictive approach and did not fully distribute its resources to external users. Instead, it emphasized conducting research through collaborative partnerships:

If we were to deal out completely, we would probably have to include that in our explanations to patients, […] What we explain to patients is that we utilize it for future patients, for future research, and [NAME of institution] is keeping an eye on what research it is used for, and we will never take a string from [NAME of institution] (PA).

Underlying this statement was a concern that freely sharing biospecimens and data outside the institution could blur the biobank’s responsibility to donors. Only one institution provided biospecimens to overseas researchers. A participant from this institution noted that, at international academic meetings, they had encountered both critical comments from abroad and strong demand for access to Japanese biospecimens:

At a biobank conference held two years ago, Japan was criticized because Japanese samples were not easily accessible to the outside world […] On the other hand, Japanese samples are very popular. […] I asked if this is because there is little variation in the genetic background, and I was told that this is partly true, but what is even more important is the fact that the lifestyle of the Japanese people is very similar (PB).

With the exception of this institution, overseas distribution was generally not permitted. While this was not explicitly stated, the findings suggest that, as in the case of provision to commercial entities discussed earlier, similar concerns about ethics committee caution and donor acceptance may have influenced decisions regarding international sharing.

Donor information may potentially be used to facilitate recruitment for other studies. One participant suggested that a subset of donors may be particularly engaged and willing to participate in a range of research activities, and that such cohorts may also attract interest from industry partners.

And then, what you mentioned earlier—what we call an “add-on cohort” — that kind of thing basically allows us to conduct additional surveys with a cohort consisting of a specific population whose health profiles have already been fairly well characterized. If we tried to do that from scratch, it would probably take something like a hundred times more money and effort. But with an existing cohort, sometimes just adding, for example, a single sheet of questionnaire data can really enrich the dataset. So in that sense, that’s part of the appeal, and it’s also something that companies, in particular, tend to show a lot of interest in (PC).

Efforts to enhance the accessibility and usability of biospecimens and data, on the one hand, facilitated expanded use by commercial entities and overseas institutions. On the other hand, they raised a range of ethical and governance challenges, including whether such practices would gain acceptance from ethics committees and donors, how responsibility toward donors should be maintained, and how consent procedures and documentation ought to be structured.

c. Stakeholders and research governance

This theme illustrated the relationships between biobanks and various stakeholders, including the public, local governments, local medical associations, patient advocacy groups, family associations, donors, and their families.

##### Public perceptions and relationship-building

This category captured public perceptions of various aspects of research and biobanking, as well as efforts to build relationships with the public in light of these perceptions. The perceptions discussed included levels of awareness and recognition of the biobank, attitudes toward genetic data and research participation, trust in biobank activities, and perceived public benefits. One participant noted that attitudes toward research participation varied depending on individuals’ affiliations. He illustrated this point by referring to patients who attended a university hospital:

Basically, patients who come to university hospitals are prepared for this. For example, there are few people who would refuse to have the student see them during practical training, and they are willing to accept the student. I feel that the hurdles to providing such specimens for use in research would also be low (PD3).

In the case of a population-based biobank that collected biospecimens from local residents, the importance of trust was emphasized as essential to long-term sustainability. One participant noted that *“in obtaining the consent of people who live in the area, and especially in the context of a long-term study, it is crucial to earn their trust”* (PC). The same participant further explained that the trust accumulated within the university’s surrounding community contributed to facilitating collaboration in research activities.

##### Medicine and health of the public, and relationship-building

Some biobanks discussed potential incentives for donors and considered what might be most effective in encouraging participation. Health-promoting measures, such as providing medical examinations, were identified as one such incentive and were regarded as particularly important:

Not only for us, but especially for population-based programs, those targeting the healthy general public, it will be very important to provide incentives for participants to benefit their health (PC).

However, such health promotion activities were described as far from straightforward, as they require the establishment of collaborative relationships with local hospitals.

##### Relationship-building with local government and medical associations

As discussed above, public trust has been identified as an essential component of sustainable biobanking in the context of community-based biobanks. In addition, local governments and medical associations were described as playing an important role in fostering trust between the public and biobanks. One participant explained that trust established between the public and local government extended to the biobank, forming what was described as a “circle of trust”:

When there is a limit to the direct approach [to the residents], when we ask ourselves who are the most trusted people by these people […] the local government is a representative public institution involved in the daily lives of its residents; then, the trust of those residents is very important for them too, so in a sense, they do not associate with strange groups, […] so, in obtaining trust [from the residents], we are kind of screened by an already trusted body (PC).

##### Relationship-building with patient advocacy groups and family associations

Patient advocacy groups and family associations were described as important actors, particularly in recruiting patients for disease-specific biobanks. Donors were recruited for biobanking through a variety of channels, including newsletters, meetings, seminars, and public lectures organized by or in collaboration with these associations:

[…] we had occasions to explain our program in places where everyone gathered, and they put up flyers at vocational aid centers. Also, […] about once a year, when holding a seminar or a public lecture […] in the community, we receive a slot for the bank at the end of the seminar and participants immediately join us (PB).

##### Donors and research governance

This category encompassed codes related to research regulation that are closely connected to donors from ethical and social perspectives. The informed consent process and associated procedures, including different consent models, constituted the primary focus of this category. All institutions implemented broad consent; however, some also considered tiered or dynamic consent models alongside broad consent.

One facility adopted a tiered approach to blood collection, allowing individuals to choose among three options: (1) collection of residual blood only after testing or diagnosis; (2) additional blood collection for biobanking at the time of testing or diagnosis; and (3) blood collection exclusively for biobanking purposes. In addition, this facility accommodated individual preferences regarding the types of materials collected. However, this tiered approach did not extend to the scope of data or sample sharing. Another institution likewise chose not to adopt a tiered consent model for sharing, citing concerns that such an approach could lead to overly complex procedures:

We considered [tiered consent], but we did not have enough time to get to that point. The other aspect was that we wanted to avoid as many complications as possible; thus, the consent itself was a choice between two options in a sense: to participate or not to participate (PC).

One participant advocated the use of dynamic consent in combination with broad consent, a framework that effectively corresponds to meta-consent.

I think it would be good to have both the dynamic and the broad consent. If you are very involved and can exchange information closely with us, then we can do such intensive research together. I think there are also many people who are not looking for such a close relationship […] I feel that it would be good to combine the two (PB).

By contrast, another participant expressed reservations about the feasibility of implementing dynamic consent, particularly from the perspective of maintaining data integrity.

Dynamic consent is not bad, but as you probably know, I feel that it is very weak in terms of so-called data integrity (PC).

Broad consent was also discussed in relation to several mechanisms aimed at enhancing the robustness of biobank systems, including information disclosure and prior ethical review.

Beyond informed consent, the return of results emerged as one of the most frequently discussed topics. This issue was addressed in multiple contexts, including whether incidental or secondary findings should be returned, how such possibilities should be described in informed consent materials, the relationship between research findings and clinical practice, and the attitudes of patients or research participants:

[If we were to return the results,] we would have to explain both the right to know and the right not to know, […] whether they want to know information about hereditary tumors related to cancer or not, […] whether we can tell their families or not, and if so, who we can tell […] if it is possible to include them in the broad consent form; so, this would be quite a challenge (PA).

What we are facing now is the issue of whether or not to inform the family about the incidental and secondary findings of the hereditary tumor after the death of the participant, which is a very sensitive and important issue (PC).

The provision of health check-ups, screenings, or other medical examinations as incentives for donors raises particular concerns about therapeutic misconception.

##### Family and research governance

This category encompassed codes related to donors’ families and their connections to research governance. Specifically, it addressed concerns regarding the collection of personal information about donors’ family members and the involvement of families in processes related to the return of results and the withdrawal of consent:

For example, there are cases where patients consent to a research project, but when they return home, their families or relatives say that the research is outrageous and that they will be used as guinea pigs, and the participants withdraw their consent. There are many cases like that. Even if the patient gives consent, it may be withdrawn later because of the family’s opinion (PB).

## Discussion

Interviews were conducted with four Japanese biobanks of varying types and sizes, and the resulting transcripts were analyzed using inductive thematic analysis. Through this process, three main themes were identified: organizational management and governance; expansion of the scope of utilization and improvement of utilization rate and efficiency; and stakeholders and research governance.

### Management of biobanks

The first theme focused on organizational management and governance aimed at sustaining biobank research infrastructure, encompassing discussions of risk management, quality management, and financial considerations. Risks such as information leakage, health hazards, and the commercialization of biospecimens were identified as challenges to organizational sustainability. In addition, references to international standardization as a form of quality management emerged in participants’ accounts, particularly in relation to the growing internationalization of biobank research (Bernasconi et al., 2020; Gille et al., 2020).

Funding was a major concern for all of the biobanks studied, consistent with findings from previous research (Simeon-Dubach and Henderson, 2014; Watson et al., 2014). As a participant notes, even if public funds are invested in a biobank when it is established, it does not necessarily mean that it will continue. While it is easy to terminate a research project, it is difficult to terminate a biobank. There are issues with the management of biospecimens and data that had been collected, or what would happen to the relationships with stakeholders and the promises made to them. A participant’s comment, “the reality is that we have made several decisions based on the need to reduce running costs” is a candid opinion from a person who has been involved in the management of the biobank. Financial soundness and independence are substantially important from the viewpoint of the maintenance and development of research infrastructure. On the other hand, if financial problems become serious, it is conceivable that the biobank could be affiliated with, for example, a foreign pharmaceutical company. In such a case, it will be necessary to consider whether the original wishes of donors, families, patients, and residents will not be damaged. The challenge, then, is to strike a balance so that measures to improve financial soundness do not run counter to the original goals and desires of donors and other stakeholders. It should be noted that the use of biospecimens and data and their efficiency, discussed below, are essential to achieving the Biobank’s goals and that financial soundness is one of the driving forces behind the provision of biospecimens and data to commercial entities, particularly.

### Effective use of biobanks

The second theme concerned the use and efficiency of biospecimens and data, which are closely linked to the advancement of biomedical research—the central objective of biobanking. A key issue within this theme was the external provision of biospecimens and data. This concern arises against the backdrop of an increasing number of reports highlighting the underutilization of biobank materials (Henderson et al., 2019; Paradiso et al., 2018).

Across all of the biobanks included in this study, the external provision of biospecimens—particularly to commercial entities—was described as a critical issue with ethical implications and consequences for donor relations. Participants consistently indicated that this issue exerted a substantial influence on institutional policies, governance arrangements, and operational practices. As illustrated by one case discussed above, one institution chose to avoid sharing biospecimens and data with external institutions, instead limiting access to materials to collaborative research contexts. From the perspective of utilization and efficiency, such restrictions may be viewed as a limiting factor. Nevertheless, this framework was adopted based on the belief that it represents the most appropriate means of safeguarding the ethical integrity of biobank research.

Thus, while the external provision of biospecimens and data enables biobanks to realize their full potential and fulfill their role in advancing biomedical research, it is simultaneously perceived as a risk factor that may undermine ethical standards and public trust. This risk is one that biobanks must actively manage, given that public trust in commercial companies is generally lower than trust in public institutions, and that donors are often motivated by altruism and expect their materials to be used for the benefit of society. Previous studies have reported that a segment of the public regards the donation of biospecimens to commercial entities as unacceptable (Critchley et al., 2021; Haddow et al., 2007). However, qualitative analyses of deliberative events grounded in deliberative democracy theory have shown that participants who were initially strongly opposed to the commercialization of biobanks became more accepting of such practices following structured deliberation (Nicol et al., 2016). The provision of biospecimens and data to commercial entities, and the broader issue of commercialization, therefore require careful ethical consideration. In light of these findings, it is necessary to develop appropriate governance measures for external provision under broad consent frameworks.

### Stakeholder engagement in biobanking

The third theme focused on the development of stakeholder relationships that are essential for obtaining donor consent, as well as the forms of research governance associated with these relationships. Because this interview study included biobanks of different types and sizes, the stakeholders involved varied across institutions, as did their respective roles. For example, a population-based biobank operating within a specific region sought to build trust and secure cooperation from local residents by working closely with the local government and medical associations. In contrast, for a disease-specific biobank, patient self-help groups and family associations emerged as particularly important stakeholders, prompting various efforts to establish and maintain relationships with these groups.

This is because no biobank project can be sustained without the cooperation of residents and patients. Participants in this interview study consistently suggested that such cooperation was fostered through two key mechanisms: trust and incentives. Indeed, the strategies described by participants were largely grounded in these relational concepts of trust and reciprocity. One strategy involved indirectly gaining the trust of potential donors by cultivating relationships with external stakeholders who were already trusted within the community. Another strategy entailed providing donors with tangible and direct benefits in return for their contributions.

While these approaches may be effective in supporting the sustainability of biobanks, the interview data also suggest that they can be interpreted as forms of relationship-building primarily aimed at attracting donors, rather than at substantively enhancing stewardship or participatory governance. In this sense, such practices may be critically understood as instrumental forms of relationship-building. As Petersen (2007) argues, stakeholder engagement framed in this way functions as a strategy of risk management, rather than as a form of genuine stakeholder “participation.”

Another important perspective on this issue concerns governance in relationships with stakeholders. Governance in relation to donors, who are central to biobanking, has traditionally been discussed primarily in terms of procedures related to informed consent, the return of results, and therapeutic misconception. With respect to informed consent, participants emphasized the importance of avoiding overly complex procedures, ensuring comprehensibility, and linking consent processes to information disclosure and ethical review mechanisms.

Regarding dynamic consent, both disease-specific and population-based biobanks expressed generally positive views, although concerns were also raised about potential implications for data integrity. Previous research has reported that donors often desire the return of results, including incidental and secondary findings (Rachul et al., 2012). In contrast, participants in this interview study tended to frame the return of results primarily as an incentive for donors. One participant, in particular, expressed concern about whether hereditary incidental or secondary findings should be disclosed to a donor’s family after the donor’s death, suggesting an awareness of the ethical duties and responsibilities borne by researchers in such situations.

Several participants also raised concerns about therapeutic misconception in relation to the return of results. However, within the context of this study, therapeutic misconception was not regarded as a major issue, as participants considered that the potential benefits of returning results outweighed the associated risks, and that misunderstandings could be mitigated through careful and sufficient explanation.

In addition, the interview findings suggest that decision-making by donors involved in biobank research is not necessarily conducted solely at the individual level. One interviewee noted that it is not uncommon for individuals who initially consent to participation to later withdraw their consent after discussing the decision with family members. This observation highlights the relational dimensions of decision-making, particularly in sociocultural contexts where health-related choices are embedded within family networks. Concerns expressed by participants often extended beyond individual privacy risks to potential implications for relatives, such as the unintended disclosure of hereditary information.

Consistent with this observation, surveys of the general public in Japan have suggested that when considering participation in biobank research, potential harms to family members may be perceived as more significant than risks to oneself, such as personal privacy invasion or individual discrimination (Oikawa et al., 2023). These tendencies align with theoretical discussions of relational autonomy, which emphasize that autonomy is not exercised in isolation but is socially embedded and shaped through interpersonal relationships (Dove et al., 2017).

### Tensions between scientific advancement and ethical practice in biobanking

The three themes generated through this qualitative research represent the underlying rationale for the diverse activities associated with biobank operations from the perspectives of biobank management. These themes can be figuratively interpreted as corresponding to the foundation, roof, and pillars of a “biobank building.” The foundation represents organizational management and governance, which enable the biobank to function as a research facility and infrastructure. The roof symbolizes activities related to the utilization of biospecimens and data for scientific advancement. The pillar supporting the development of biobank research represents engagement activities that build relationships with society. Together, these elements illustrate ongoing efforts to advance biobanking as a scientific enterprise while ensuring that its development remains ethically grounded within broader social practices. Taken together, these dimensions may be understood as reflecting ongoing tensions between scientific advancement, institutional governance, and societal engagement, corresponding respectively to the “roof,” “foundation,” and “pillar” elements of the biobank metaphor.

While the fundamental goals of biobanking—facilitating biomedical research through the long-term storage of biospecimens and associated data, and their subsequent use—are broadly shared across countries, governance frameworks differ across jurisdictions, including in their legal foundations. In some Asian contexts, such as Taiwan and Singapore, dedicated legislation directly regulates biobanking activities, notably the *Human Biobank Management Act (HBMA)* in Taiwan and the *Human Biomedical Research Act (HBRA)* in Singapore, which explicitly codify consent procedures, benefit-sharing arrangements, and penalties for non-compliance (Chalmers et al., 2016). By contrast, in Japan, governance has historically tended to rely more on a combination of general statutory regulation and detailed ethical guidelines rather than a dedicated biobank-specific legal framework. In particular, practices surrounding biospecimen and data management are shaped by the *Act on the Protection of Personal Information* alongside the *Ethical Guidelines for Medical and Biological Research Involving Human Subjects*. Rather than constituting a dedicated biobank law, this combination functions as a relatively flexible regulatory framework, which may contribute both to cautious practices—particularly regarding international data sharing and collaboration with commercial entities—and to greater variability in governance approaches across institutions.

As discussed earlier, participation decisions in biobank research within the Japanese context often reflect relational considerations, including potential implications for family members. Concerns about stigma, discrimination, or the disclosure of hereditary information may extend beyond the individual participant, suggesting that consent decisions may be embedded within family and social relationships rather than framed purely as individual acts of autonomy. Such patterns resonate with relational understandings of autonomy and highlight how sociocultural contexts shape both participation decisions and governance expectations. By contrast, Western biobank governance—particularly in Europe and North America—has historically tended to emphasize individual autonomy as a primary ethical foundation of informed consent. Regulatory frameworks such as the *Federal Policy for the Protection of Human Subjects (Common Rule)* in the United States and governance structures influenced by the *General Data Protection Regulation (GDPR)* in Europe tend to conceptualize consent primarily as an individual legal and ethical act (Hallinan, 2020; Lynch et al., 2019). While this emphasis facilitates standardized consent procedures and international data-sharing infrastructures, it may not fully capture relational dimensions of decision-making that appear more salient in some sociocultural contexts.

Another cross-cutting issue emerging from our interviews concerns the role of trust in shaping relationships between biobanks and society. Trust was frequently invoked by participants as an essential condition for sustaining participation and institutional legitimacy, consistent with prior theoretical and empirical work identifying trust as central to biobank governance (Kraft et al., 2018; Nielsen and Kongsholm, 2022; Samuel et al., 2022a). However, trust was not always articulated as a normative framework for stakeholder engagement aimed at enhancing the *trustworthiness* of governance. Rather, the limited data from this study suggest that biobank operations tended to function less as mechanisms for strengthening intrinsic trustworthiness and more as instrumental or risk-management strategies designed to secure broader social trust. As Samuel et al. (2022a) caution, it is essential to ensure that efforts to maintain public trust do not come at the expense of acting in a genuinely trustworthy manner.

Taken together, these findings suggest that tensions between scientific advancement and ethical practice in biobanking cannot be understood solely in technical or procedural terms. Instead, they are deeply shaped by regulatory cultures, legal traditions, sociocultural understandings of autonomy and family responsibility, and evolving expectations regarding trust and public engagement. Recognizing these contextual differences will be essential for developing internationally interoperable governance models that remain scientifically enabling while ethically robust. Incorporating relational perspectives on autonomy and critically engaging with the role of trust in governance may contribute to more culturally responsive consent processes and to the cultivation of sustainable trust in global biobank collaborations.

### Limitations

This study has several limitations. Although participants were recruited from different types of biobanks in an effort to avoid uniformity of perspectives, the number of participating institutions was small and constrained by contextual factors rather than by theoretical sampling considerations. As a result, interviews were conducted with only four biobanks, which is insufficient to capture the full diversity of views within the Japanese biobanking landscape. Moreover, all participating institutions employed broad consent, which limited the availability of empirical insights into alternative consent models, such as dynamic consent. Consequently, the range and diversity of perspectives represented in this study were constrained. Nevertheless, *post hoc* analysis indicated that data saturation was achieved for the key analytical categories, supporting the adequacy of the sample for the exploratory aims of the study.

The interview data were analyzed using thematic analysis, which may also entail methodological limitations. While thematic analysis is recognized for its flexibility compared with other qualitative methods, this same flexibility has been noted as a potential source of reduced consistency in the development of themes from qualitative data (Nowell et al., 2017). Accordingly, the analysis presented here cannot entirely rule out this limitation inherent to the use of thematic analysis.

## Conclusion

This study aimed to elucidate how biobank governance is constructed and what the current challenges are through the experiences of those directly involved in operating biobanks. Qualitative interviews were conducted with managers of biobanks of different types and scales in Japan. Analysis of the resulting statements generated three themes related to sustainable biobank operation, the efficient utilization of biospecimens and information, and stakeholder engagement. These themes reflect the tension that exists in the operation of biobanks as medical research infrastructure between scientific advancement and ethical practice. It was within this tension that governance was conceived and practiced. Against the backdrop of a regulatory environment in Japan that provides limited concrete guidance, this study has shed light on how individual institutions have sought to promote the role of biobanks as scientific research infrastructure while simultaneously attempting to realize ethical practices in context-specific ways. Moreover, these efforts reveal the practical processes through which governance has been negotiated and enacted across institutions.

At the same time, the findings indicate that many of these governance practices were primarily oriented toward managing practical constraints and sustaining operations, rather than explicitly engaging with broader normative questions, such as what constitutes trustworthy governance, how accountability and transparency should be conceptualized, and whose values ought to be reflected in decision-making. Taken together, these findings suggest that biobank governance may benefit from moving beyond purely instrumental approaches and from further articulating normative foundations to support sustainable practices. At the same time, given the limited scope of this study, future research is needed to explore how such normative dimensions of governance are understood and enacted across a broader range of biobank contexts. Further research would also benefit from examining the perspectives of diverse stakeholders involved in or affected by biobank activities—including researchers, ethics committee members, donors, members of the public, and industry partners—in order to better understand how expectations, values, and concerns regarding biobank governance are formed and negotiated.

## Data Availability

The raw data supporting the conclusions of this article will be made available by the authors, without undue reservation.
